# Childhood socioeconomic position and adult mental wellbeing: Evidence from four British birth cohort studies

**DOI:** 10.1371/journal.pone.0185798

**Published:** 2017-10-25

**Authors:** Natasha Wood, David Bann, Rebecca Hardy, Catharine Gale, Alissa Goodman, Claire Crawford, Mai Stafford

**Affiliations:** 1 MRC Unit for Lifelong Health and Ageing at UCL, London, United Kingdom; 2 UCL Institute of Education, London, United Kingdom; 3 MRC Life Course Epidemiology Unit, University of Southampton, Southampton, United Kingdom; 4 The Institute for Fiscal Studies, London, United Kingdom & University of Warwick, Coventry, United Kingdom; Ege University, School of Medicine, TURKEY

## Abstract

**Background:**

There is much evidence showing that childhood socioeconomic position is associated with physical health in adulthood; however existing evidence on how early life disadvantage is associated with adult mental wellbeing is inconsistent. This paper investigated whether childhood socioeconomic position (SEP) is associated with adult mental wellbeing and to what extent any association is explained by adult SEP using harmonised data from four British birth cohort studies.

**Methods:**

The sample comprised 20,717 participants with mental wellbeing data in the Hertfordshire Cohort Study (HCS), the MRC National Survey of Health and Development (NSHD), the National Child Development Study (NCDS), and the British Cohort Study (BCS70). Warwick Edinburgh Mental Wellbeing Scale (WEMWBS) scores at age 73 (HCS), 60–64 (NSHD), 50 (NCDS), or 42 (BCS70) were used. Harmonised socioeconomic position (Registrar General’s Social Classification) was ascertained in childhood (age 10/11) and adulthood (age 42/43). Associations between childhood SEP, adult SEP, and wellbeing were tested using linear regression and multi-group structural equation models.

**Results:**

More advantaged father’s social class was associated with better adult mental wellbeing in the BCS70 and the NCDS. This association was independent of adult SEP in the BCS70 but fully mediated by adult SEP in the NCDS. There was no evidence of an association between father’s social class and adult mental wellbeing in the HCS or the NSHD.

**Conclusions:**

Socioeconomic conditions in childhood are directly and indirectly, through adult socioeconomic pathways, associated with adult mental wellbeing, but findings from these harmonised data suggest this association may depend on cohort or age.

## Background

Good mental wellbeing is an area of increasing importance for society, as those with high levels of wellbeing have been shown to have greater longevity [1] [2] and a lower risk of developing certain chronic diseases [3]. Wellbeing has also been identified as a key component of healthy ageing [4] [5] and given the ageing population, it is becoming increasingly important to understand the determinants of good mental wellbeing at mid to later life. Positive mental wellbeing is multidimensional, incorporating hedonic (e.g. life satisfaction and positive affect) and eudemonic (e.g. positive psychological functioning) perspectives [2].

Evidence has shown that proximal factors in adulthood, such as socioeconomic and psycho-social circumstances, are associated with mental wellbeing [2]. However, more distal factors, such as socioeconomic circumstances experienced in childhood, and their links to wellbeing in adulthood are not so well understood, despite a growing body of life course research demonstrating that childhood socioeconomic circumstances are associated with a range of factors in adulthood including socioeconomic circumstances [6] and physical and mental health [7] [8] [9].

The evidence on childhood socioeconomic position (SEP) and adult mental wellbeing is mixed. Two studies, both using data from the Survey of Health, Ageing and Retirement (SHARE), found an association between more disadvantaged childhood SEP and lower life satisfaction at older ages (50 years and older) that persisted after adjusting for adult circumstances, such as education, income, employment and partnership status [10] [11]. One study of Australian baby-boomers in the early sixties [12] and one of people in their mid-30s living in Britain [13] have found that the association between childhood SEP and adult wellbeing was fully attenuated by adjustment for adult SEP, thus suggesting that socioeconomic disadvantage in childhood sets the course for a disadvantaged trajectory during adulthood which can then affect wellbeing [14].

Other studies have found no association between childhood SEP and adult mental wellbeing. One study using prospective cohort data from the MRC National Survey of Health and Development (NSHD) found that childhood SEP, measured through father's occupational class; parental education; lack of household amenities; crowding at birth and having a teenage mother or father at birth, was neither directly, or indirectly through adult socioeconomic circumstances, associated with adult mental wellbeing at age 60–64 years [15]. This is in accordance with an earlier, smaller study, using retrospective childhood SEP data from the Boyd-Orr cohort in Britain [16], which also found that there was no association between childhood SEP and quality of life in later adulthood (during late 60s and 70s), but quality of life was instead associated with more proximal circumstances, such as adult material resources and health.

The inconsistencies in the current evidence could be due to measurement differences, either in the different measures of wellbeing which have been utilised, or in the measurement of childhood or adult SEP. The association might also depend on national context, age or cohort. We are not aware that age or cohort differences in the association between childhood SEP and wellbeing have been tested but note that the evidence from Britain indicates an association between childhood SEP and adult wellbeing among those born in 1970 [13] but not those born in 1946 [15] or 1930s [16]. Plausibly, the social changes which occurred in Britain during the latter half of the twentieth century, such as increasing social inequality and large gains in the average standard of living [17], might modify associations. Even when SEP is sufficient to meet basic needs, the relative disadvantage of not being able to live up to societal expectations and having relatively lower levels of income may be detrimental to wellbeing [18] [19]. Differences between previous studies might also be because wellbeing was measured at different ages (ranging from 34 to 65 years and older). Again, we are not aware of studies that have tested whether an association between childhood SEP and wellbeing weakens or strengthens through adulthood.

The aims of this paper are to examine the association between childhood SEP and adult mental wellbeing and to determine to what extent the association was mediated by adult SEP, using harmonised prospective data from four British cohorts to minimise differences due to measurement. We additionally aim to explore whether associations differ across these cohort studies (between 1930 and 1970), but we note that age and birth cohort effects cannot be differentiated here.

## Methods

### Data

Data from four British birth cohort studies were used. The Hertfordshire Cohort Study (HCS) comprises 3,000 people, born between 1931 and 1939, who were still residing in the county of Hertfordshire in the 1990s [20]. The MRC National Survey of Health and Development (NSHD) comprise 5,362 people born in one week in March 1946 in Britain from non-manual and agricultural occupation families and a random one in four sample of births from families of manual occupation [21]. The National Child Development Study (NCDS) comprises all 17,415 people born in one week in Britain in 1958 [22] and the British Cohort Study (BCS70) comprises all 17,196 people born in one week in Britain in 1970 [23].

Ethical approval has been granted for the four studies. The HCS has ethical approval from the Hertfordshire and Bedfordshire Local Research Ethics Committee and all participants have given informed consent. The NSHD was granted ethical approval from the Greater Manchester Local Research Ethics Committee and the Scotland A Research Ethics Committee and all participants have provided informed consent. The NCDS and the BCS70 have been granted ethical approval for each sweep from 2000 by the National Health Service (NHS) Research Ethics Committee and all participants have given informed consent.

### Wellbeing

Wellbeing was measured using the Warwick-Edinburgh Mental Wellbeing Scale (WEMWBS), which was administered via a self-completion paper questionnaire. WEMWBS is a validated instrument, which combines both hedonic and eudemonic elements of wellbeing [24]. WEMWBS consists of 14 separate items measured on a 5 point Likert scale which are summed to give a total score of wellbeing ranging from 14 to 70, a higher score reflecting higher levels of wellbeing. Those who were missing 3 items or less were imputed scores which reflected the overall mean score of the items for which they had data. In the HCS wellbeing was measured at age 73, in the NSHD at age 60 to 64, in the NCDS at age 50 and at age 42 in the BCS70. Unfortunately, as WEMWBS was not collected at the same age in the four cohorts consequently we were unable to investigate age or cohort effects. Cronbach’s alpha test of reliability showed high levels of internal consistency (0.91 in all cohorts) for the WEMWBS measure.

### Childhood socioeconomic position

Childhood SEP was measured through father’s occupational social class which was collected prospectively at ages 10 to 11, with the exception of the HCS, where father’s occupation at birth was reported retrospectively when participants were aged 59 to 72 years. Occupational social class was coded according to the Register General’s classification, where class is defined according to job type. Job type was measured using the Office for National Statistics Standard Occupational Classification 1990 (SOC90) [25] for the HCS, NCDS and BCS70, and 1970 (SOC70) for the NSHD [26]. For those cases which were missing the Register General’s classification of father’s social class using SOC90 in the NCDS (*n* = 1,232) and the BCS70 (*n* = 610), the Register General’s classification was derived using SOC70 in the NCDS and SOC80 in the BCS70, instead. SOC70 and SOC80 are both comparable to SOC90, but a sensitivity analysis was run with these SOC70 and SOC80 substitutions excluded and similar results were found (S1 Table). More information on the Standard Occupation Classification (SOC) coding is provided by the UK Office for National Statistics: https://www.ons.gov.uk/methodology/classificationsandstandards/standardoccupationalclassificationsoc

### Adult socioeconomic position

Adult SEP was measured using education and occupational social class. Education was measured using age left continuous full-time education and occupational social class was measured using the Register General’s classification based on SOC90 for all four cohorts. In the NSHD, NCDS and the BCS70 adult occupational social class was based upon the study member’s own social class, whilst in the HCS women who were married were assigned the social class of their husband. Adult social class was measured between the ages of 60 to 68 years (1994) in the HCS. For those who had retired in the HCS the respondent’s (or husband’s) most recent job was used to derive social class. Adult social class was measured at midlife in the NSHD (age 43, in the year 1989), NCDS (age 42, 2000) and the BCS70 (age 42, 2012) as these measures were harmonised as part of a previous work package [26]. Where adult social class was missing we used occupational social class data collected at age 53 years (1999, *n* = 150) in the NSHD, at ages 46 (2004, *n* = 53) or 50 years (2008, *n* = 933) in the NCDS and in the BCS70 at age 38 (2008, *n* = 316).

### Covariates

Additionally, we adjusted for a number of covariates in adulthood which have been shown to be associated with adult mental wellbeing. These were measured at the same age as wellbeing and comprise reporting a long-term limiting illness, partnership status and sex [2].

### Statistical methods

We derived ridit scores for each social class category calculated as the proportion of the sample with a more disadvantaged social class, plus half the proportion in that category. The score thus ranged from 0 (highest social class) to 1 (lowest social class). In linear regression analysis the ridit score coefficient is the slope index of inequality (SII), interpreted as the absolute difference in wellbeing between the top and bottom of the social class distribution. This technique takes account of the population distribution across social class categories, enabling comparison over time [27] [28] and assumes a linear association between the outcome and social class. We found no evidence of non-linearity in the association between father’s or adult social class and wellbeing (results shown in S2 Table).

Linear regression analysis was carried out using STATA 14 to examine associations between wellbeing and i) father’s social class, ii) adult social class, iii) father’s and adult social class included together. Data from the four cohorts were pooled. Main effects for cohort and interaction terms for cohort by social class were also included with BCS70 as the reference category. The BCS70 was used as a reference category as it had the largest sample size.

We then tested whether any associations between wellbeing and father’s social class were mediated by own education or adult social class using multi-group path analysis. The multi-group option allows cohort differences to be tested by comparing a model where the parameters representing direct and indirect (through education and adult social class) effects are constrained to be equal across the cohorts to a model where they are unconstrained. Model fits were evaluated using goodness of fit statistics: *x*^2^; the root mean square error of approximation (RMSEA); the comparative fit index (CFI); and the Tucker Lewis index (TLI). The path analysis was conducted using Mplus v7.

Preliminary models indicated there was no sex difference in the association between father’s social class and wellbeing (data available from the authors). We present regression models adjusted for sex, long-term limiting illness and partnership status in the main text and sex only in S3 Table. Compared with the sex only adjusted models, the majority of estimates were partly attenuated in the models which additionally adjusted for long-term limiting illness and partnership. Estimates were similar and the same estimates attained statistical significance in both sets of models, with two exceptions. Mean difference in wellbeing between NCDS and the reference group (BCS70) was greater in the fully-adjusted model than in the sex-adjusted model. In addition, the positive mean difference in wellbeing between women compared with men was greater in the fully-adjusted models. In other words, limiting illness and partnership status suppressed some of the differences in mean wellbeing between genders and cohorts.

### Analytic sample

The analytic sample comprised cases with complete data on wellbeing (n = 1,402 in the HCS, n = 1,978 in the NSHD, n = 8,745 in the NCDS and n = 8,589 in the BCS70). A comparison of the cases which had complete data and those that were missing wellbeing data was carried out, which is detailed in the supplementary information (S4 and S5 Tables). Overall cases with missing wellbeing data were more likely to be male, more likely to have had fathers who were in a lower social class, had fewer years of education, more likely to be in a lower adult social class, more likely to be unpartnered and have a long-term limiting illness, than those cases which had wellbeing data.

Full information maximum likelihood estimation was used (FIML) to account for those with missing data on father’s social class, education, adult social class, partnership status or long-term limiting illness. Full information maximum likelihood (FIML) estimation assumes that those data were missing at random and uses all the information which is available in the data to model estimates. FIML has shown to be less biased than listwise deletion, or complete case analysis [29].

## Results

### Sample characteristics

Table 1 shows that mean wellbeing was highest for those in the HCS, 51.9, and lowest in the NCDS, 49.2, and the BCS70, 49.1 (*p*<0.05), this is in line with previous research which has found that wellbeing is higher among older age groups [1]. The distribution of father’s social class differed among the cohorts with manual occupations predominating in the earlier born cohorts, but less so in the later born cohorts (Table 1). Those in younger born cohorts were more educated, more likely to be in an intermediate occupation in adulthood and less likely to have a long-term limiting illness than earlier born cohorts.

**Table 1:** Sample characteristics across the four birth cohorts.

	HCS	NSHD*	NCDS	BCS70
*Birth year*	*1931–39*	*1946*	*1958*	*1970*
*Year WEMWBS measured*	*2003–05*	*2006–09*	*2008*	*2012*
*Age WEMWBS measured*	*73*	*60–64*	*50*	*42*
	**% / Mean (SD)**	**% / Mean (SD)**	**% / Mean (SD)**	**% / Mean (SD)**
**Wellbeing; mean (SD)**	51.9 (8.1)	51.6 (8.3)	49.2 (8.1)	49.1 (8.3)
**Sex**				
Male	50.7	46.7	48.2	47.0
Female	49.3	53.3	51.9	53.0
**Father's social class**				
I (Professional)	1.1	3.9	5.2	5.5
II (Intermediate)	8.5	15.8	21.3	26.1
III (Skilled Non-Manual)	8.9	10.1	11.3	11.1
III (Skilled Manual)	46.9	45.7	40.5	38.4
IV (Partly skilled)	25.8	17.7	14.1	13.0
V (Unskilled)	8.8	6.8	7.6	5.8
**Age left full time education; mean (SD)**	15.5 (1.5)	16.5 (2.4)	18.4 (5.6)	18.4 (3.4)
**Adult social class**				
I (Professional)	7.4	5.8	5.6	6.4
II (Intermediate)	27.7	36.6	38.5	44.9
III (Skilled Non-Manual)	12.2	23.7	21.9	18.8
III (Skilled Manual)	36.0	18.4	18.7	16.2
IV (Partly skilled)	13.8	12.0	12.2	11.8
V (Unskilled)	2.9	3.4	3.0	2.0
**Partnership status**				
Partnered	80.8	83.3	80.1	79.5
Unpartnered	19.2	16.7	19.9	20.5
**Long-term limiting illness**				
No	66.3	73.8	84.8	71.3
Yes	33.7	26.2	15.2	28.7
***N***	*1402*	*1978*	*8745*	*8589*

* NSHD descriptive statistics are weighted to account for the stratified sampling design

### Associations between childhood SEP, adult SEP and adult mental wellbeing

Model 1 in Table 2 shows the association between father’s social class and adult mental wellbeing, not adjusted for adult social class. The model comprises the main effects for father’s social class on wellbeing for the BCS70 (as the BCS70 is the reference category) and an interaction between father’s social class and cohort, to show the association for the other cohorts. In the BCS70 there was an association between father’s social class and adult mental wellbeing and those whose fathers were at the bottom of the social class distribution (ridit score of 1) had a mean wellbeing score which was 2.341 lower (95% CI: -3.00, -1.68) compared to those whose fathers were at the top of the social class distribution (ridit score of 0). This association was smaller in magnitude in the other three cohorts, which is shown by the positive interaction terms between father’s social class and cohort (though the interaction between cohort and father’s social class did not attain statistical significance in the NSHD or the HCS, possibly because of smaller sample sizes). For example, in the NCDS the association interaction term was 1.065 (95% CI: 0.118, 2.012) so that each 1 unit increase in father’s social class ridit score is associated with a 1.276 point lower wellbeing score (-2.341+1.065). Model 2 (Table 2, not adjusted for father’s social class) shows the association between adult social class and adult mental wellbeing, with an interaction term between adult social class and cohort. In three of the cohorts those who were at the bottom of the adult social class distribution (ridit score of 1) had lower levels of wellbeing than those at the top of the social class distribution (ridit score of 0), but this was not evident in the HCS. The interaction term for the HCS was 3.043 (CI: 1.360, 4.726) showing that both those who were at the top and the bottom of the adult social class distribution in the HCS had comparable levels of wellbeing.

**Table 2:** Associations between adult mental wellbeing and social class in childhood and adulthood.

	Father’s social class (model 1)		Adult social class (model 2)		Father's social class and adult social class (model 3)	
	Coef	SE	Coef	SE	Coef.	SE
**Father's social class (ridit score)**^a^	-2.341**	0.337			-1.763**	0.343
Cohort*father's social class (ref: BCS70*father's social class) ^a^						
NCDS	1.065*	0.483			1.203*	0.493
NSHD	1.503	0.780			2.018*	0.823
HCS	1.009	0.881			0.371	0.895
**Adult social class (ridit score)**^a^			-3.291**	0.342	-3.020**	0.350
Cohort*adult social class (ref: BCS70*adult social class) ^a^						
NCDS			0.025	0.472	-0.140	0.484
NSHD			-0.020	0.788	-0.313	0.836
HCS			3.043**	0.859	3.070**	0.876
**Cohort (ref: BCS70)**						
NCDS	-0.973**	0.274	-0.437	0.269	-0.976*	0.330
NSHD	1.497*	0.441	2.247**	0.441	1.362*	0.515
HCS	2.435**	0.498	1.410*	0.492	1.184	0.607
**Sex (ref: male)**						
Female	0.267*	0.111	0.326*	0.111	0.325*	0.111
**Partnership (ref: partnered)**						
Unpartnered	-2.324**	0.140	-2.235**	0.140	-2.206**	0.140
**Long-term limiting illness (ref: no)**						
Yes	-3.747**	0.135	-3.713**	0.134	-3.679**	0.134
*Constant*	*51*.*724*	*0*.*205*	*52*.*148*	*0*.*207*	*52*.*906*	*0*.*246*
*N*	*20714*					

^a^ Father’s and adult social class is a ridit score from 0 to 1 with a value closer to 1 indicating more disadvantaged social class. Analysis carried out using linear regression.

** p<0.001

*p<0.05

Model 3 included both father’s social class and adult social class and their respective cohort interactions (Table 2). An association between father’s social class and wellbeing, independent of adult social class was found in the BCS70, but this was not as marked in the NCDS and NSHD cohorts, once adult social class was included in the model. Associations between wellbeing and social class were not materially different in the models which did not include long-term illness and partnership status as covariates (S3 Table). Sensitivity analyses indicated the substitution of SOC70 or SOC80 for those with missing SOC90 father’s social class data did not alter estimates (S1 Table). However, when analyses were limited to those with complete data on wellbeing and all covariates, the estimates of associations between wellbeing and both father’s and adult social class were considerably smaller in magnitude (S4 Table). This suggests analyses based on complete cases may be biased with respect to estimating social class differences in wellbeing.

Table 3 shows the results of the unconstrained path model. The model was a good fit to the data indicated by the CFI, TLI and RMSEA (0.965, 0.912 and 0.040 respectively) and a significantly better fit than the model which constrained the direct and indirect paths to be equal across cohorts (difference in χ^2^ 310.0 on 15 df, *p*<0.001).

**Table 3:** Path analysis coefficients for the associations between father’s social class and adult Warwick-Edinburgh Mental Well-Being scores, mediated through adult SEP.

	HCS		NSHD		NCDS		BCS70	
	Coeff	SE	Coeff	SE	Coeff	SE	Coeff	SE
*N*	*1405*		*1978*		*8745*		*8589*	
**Direct effects**								
Father’s social class^a^ -> WEMWBs	-1.570	0.841	0.101	0.794	-0.493	0.355	-1.307**	0.356
**Indirect effects**								
Indirect effect of father’s social class -> on combined adult SEP-> WEMWBs	0.045	0.180	-0.338	0.363	-0.586**	0.091	-0.778**	0.101
Father’s social class-> education	-1.174**	0.145	-4.340**	0.199	-4.726**	0.235	-3.427**	0.141
Father’s social class-> adult social class	0.139**	0.027	0.123**	0.025	0.176**	0.012	0.116**	0.012
Education^b^ -> WEMWBs	-0.039	0.153	-0.019	0.088	0.013	0.016	0.143**	0.029
Education^b^ -> adult social class	-0.055**	0.005	-0.045**	0.002	-0.012**	0.001	-0.025**	0.001
Adult social class^c^ -> WEMWBs	-0.005	0.814	-3.421**	0.827	-2.974**	0.339	-2.465**	0.377
**Model fit**								
CFI	0.965							
TLI	0.912							
RMSEA	0.040							

^a^ Father’s social class is a ridit score from 0 to 1 with a value closer to 1 indicating more disadvantaged social class;

^b^ Higher values of education indicate more years of schooling;

^c^ Higher values of adult social class indicate more disadvantaged social class

** p<0.001

**Fig 1:**
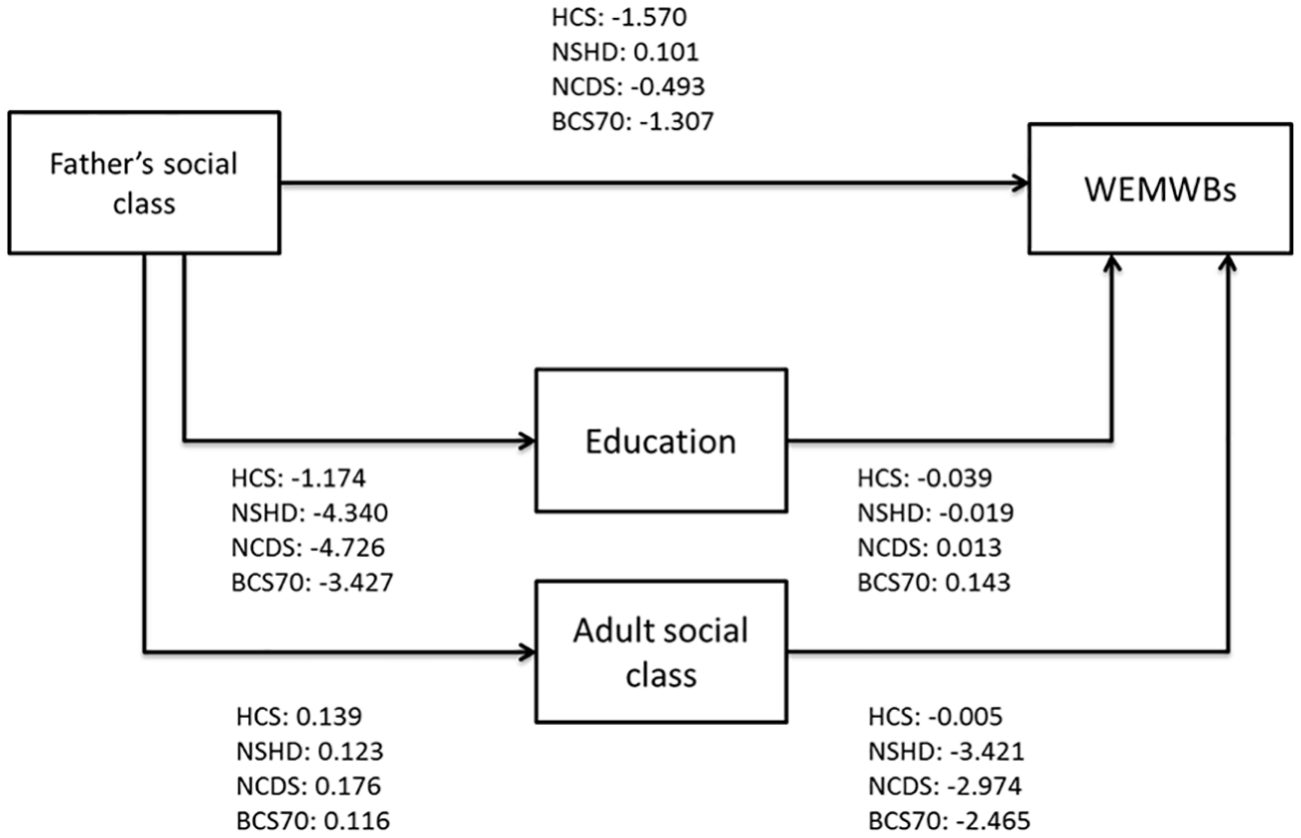
Path model of father’s social class and Warwick-Edinburgh Mental Wellbeing scores, mediated through adult SEP for the four birth cohort studies.

The path model (Table 3 and Fig 1) shows that there was a direct association between father’s social class and adult mental wellbeing only in the BCS70. There was an indirect association between father’s social class and adult mental wellbeing in the NCDS and the BCS70. Adult social class contributed to this mediation in both these cohorts, whilst education contributed only in the BCS70. Lower levels of education were associated with lower levels of wellbeing (independently of adult social class) only in the BCS70, whilst more disadvantaged adult social class was associated with poorer wellbeing in the BCS70, the NCDS and NSHD.

## Discussion

The aim of this analysis was to investigate the association of father’s social class with adult mental wellbeing and to test its consistency using harmonised, prospective data from four successive British birth cohorts. We found that a more advantaged father’s social class was associated with higher wellbeing in the two most recent cohorts (the NCDS and BCS70); these associations were linear across the socioeconomic classes. In the NCDS, this association was fully mediated by adult socioeconomic circumstances. In the BCS70, there was evidence for partial mediation through adult socioeconomic position, but higher social class in childhood was also associated with greater wellbeing, independently of adult social class.

There are a number of possible explanations for an association between father’s social class and wellbeing independent of adult socioeconomic position. One is continuity of mental wellbeing across the life course. Childhood socioeconomic advantage is associated with positive mental wellbeing in childhood, which is in turn associated with adult mental wellbeing [30–32]. Other explanatory pathways include the positive associations between childhood socioeconomic advantage and higher educational attainment, higher adult social class, and higher social engagement with friends, family and social activities, each of which are linked to more positive mental wellbeing [33]. One other possible explanation for the direct association between childhood SEP and adult mental wellbeing might be related to personality [34,35]. Evidence has shown that adverse conditions in early life, including financial conditions, can suppress optimism, which can influence future success and life trajectories. Lower optimism in adulthood has been shown to be associated with lower adult mental wellbeing [36].

Our investigation of differences in the association between father’s social class and adult wellbeing across the cohort studies was exploratory, but there are a number of plausible explanations for why this association was seen independently of adult social class in the BCS70, but not in the other studies. Firstly, the 1970s were a period of both social and economic change. The economic turbulence and decline of the manufacturing industry would have disproportionately affected those in lower social class families in BCS70, which would have resulted in greater disparities between those in the highest social classes and those in the lowest. Greater disparities between the social classes could have resulted in longer-term negative associations between childhood SEP and wellbeing due to unfavourable social comparisons in income and not being able to live up to societal expectations, which have been linked to poorer mental wellbeing [18]. There were also changes in family structure at this time, including an increase in divorce and shifts in the socioeconomic patterning of parental divorce [8]. Disrupted family relationships could affect cohort members’ levels of long-term wellbeing, not just through the economic pathways, but also through social relationships, or through psychological adjustment in adulthood [37–39], which in turn have shown to be closely associated with wellbeing [13]. These are all potential avenues for future research. An alternative explanation is a methodological one. We were unable to separate age and birth cohort effects and follow-up time since childhood and exposure to adult socioeconomic position are shorter in the BCS70, so the effects of childhood may still be pertinent for this cohort.

Previous studies have shown inconsistencies in the association between childhood SEP and adult mental wellbeing [10, 11, 12, 13, 15, 16], which could have been due to modification of this association by country, cohort or age, or because of the different measures of wellbeing and SEP utilised. Our findings used harmonised data on the wellbeing outcome and the SEP exposures and yet we still found differences across these four studies set in Britain, which is suggestive of cohort or age differences. We considered WEMWBS, which was designed to capture positive affect and positive psychological functioning. Future work might examine whether father’s SEP is related to each of these wellbeing dimensions, and others such as life satisfaction, in the same way and through the same socioeconomic pathways in adulthood.

### Strengths and limitations

There are a number of strengths and limitations to this analysis. The strengths include using a large sample size comprising four British birth cohorts, three of which were representative of the national population. The analysis used measures which had been harmonised across the four birth cohorts enabling more accurate comparisons and included WEMWBS which captures both hedonic and eudemonic wellbeing.

The analysis was limited in being unable to differentiate age and cohort differences in the associations between father’s social class and wellbeing. Additionally, despite harmonisation of measures, some methodological differences between the HCS and the other three cohorts remain. On the HCS father’s social class was measured retrospectively and adult social class was captured near to retirement (at ages 60 to 68), and therefore may not accurately reflect their main occupation and socioeconomic circumstances during their working life. Additionally, for married women in HCS, adult social class was based upon their husband’s social class, although we found no sex difference in the association between adult social class and wellbeing.

Study members who did not work (or who had a father who did not work) were not able to be assigned a social class. If they provided wellbeing data they were included in the current analyses (using a full information maximum likelihood approach, under the assumption that their data were missing at random). This may go some way towards reducing bias arising from missing socioeconomic data. However, previous research in these cohort studies has found that attrition is greater for those who had lower educational attainment, and whose fathers were in a lower social class in the NSHD [40], the BCS70 [41] and the NCDS [42], whilst the HCS has been found to be less representative of those who were at the extremes of the socioeconomic distribution [20].

In summary, this study shows that father’s social class has a long-term association with wellbeing, at least in two of the four cohorts examined, and this association was strongest in the BCS70. Both child and adult social class were related to wellbeing when mutually adjusted in the BCS70. In addition, there was evidence that father’s social class was associated with wellbeing through an adult socioeconomic pathway in the NCDS and the BCS70.

## Conclusions

These findings imply that socioeconomic conditions in childhood are important for wellbeing in mid to later life, as we found that socioeconomic conditions in childhood are directly and indirectly, through adult socioeconomic pathways, associated with adult mental wellbeing. Initiatives to improve wellbeing in adulthood should also consider providing economic support for families to improve childhood socioeconomic conditions. Future studies are warranted to examine possible cohort or age differences in the association between childhood conditions and mental wellbeing in adulthood.

## Supporting information

S1 Table Association between father's social class and Warwick-Edinburgh Mental Well-Being comparing father’s social class including SOC70 and SOC80 substitutions and father’s social class excluding substitutions. (DOCX)

S2 Table Sex-adjusted associations between Warwick-Edinburgh Mental Well-Being scores and father's social class or adult social class coded as categorical variables. (DOCX)

S3 Table Gender-adjusted associations between adult mental wellbeing and social class in childhood and adulthood. (DOCX)

S4 Table Comparison of the cases with missing data on the Warwick-Edinburgh Mental Well-Being Scale and cases with complete data on the Warwick-Edinburgh Mental Well-Being Scale. (DOCX)

S5 Table Childhood social class and adult social class associations with the Warwick-Edinburgh Mental Well-Being scores, complete cases. (DOCX)
