# Supplementary material for: Childhood socioeconomic position and adult mental wellbeing: Evidence from four British birth cohort studies
Source: PLoS One. 2017 Oct 25;12(10):e0185798. doi: 10.1371/journal.pone.0185798 (PMC5656308; doi:10.1371/journal.pone.0185798)
Supplement: S1 Table — (DOCX) [file pone.0185798.s001.docx]

S1 Table: Association between father's social class and Warwick-Edinburgh Mental Well-Being comparing father’s social class including SOC70 and SOC80 substitutions and father’s social class excluding substitutions

|  | **Father’s social class substituted with SOC70 (NCDS) and SOC80 (BCS70) for those missing SOC90** | | **Father's social class missing data on SOC90 not substituted** | |
| --- | --- | --- | --- | --- |
|  | **Coef** | **SE** | **Coef** | **SE** |
| **Father's social class (ridit score)** | -2.324^**^ | 0.335 | -2.274^**^ | 0.351 |
| **Cohort*father's social class (ref: BCS70*father's social class)** |  |  |  |  |
| NCDS | 1.058^*^ | 0.480 | 0.919 | 0.515 |
| NSHD | 1.491 | 0.773 | 1.426 | 0.780 |
| HCS | 1.001 | 0.875 | 0.930 | 0.880 |
| **Cohort (ref: BCS70)** |  |  |  |  |
| NCDS | -1.071^**^ | 0.277 | -0.937^*^ | 0.295 |
| NSHD | 1.401^*^ | 0.447 | 1.413^*^ | 0.450 |
| HCS | 2.419^**^ | 0.499 | 2.422^**^ | 0.502 |
| **Sex (ref: male)** |  |  |  |  |
| Female | 0.187 | 0.120 | 0.166 | 0.126 |
| **Partnership (ref: partnered)** |  |  |  |  |
| Unpartnered | -2.275^**^ | 0.152 | -2.191^**^ | 0.162 |
| **Long-term limiting illness (ref: no)** |  |  |  |  |
| Yes | -3.705^**^ | 0.146 | -3.581^**^ | 0.153 |
| ***Constant*** | *51.783* | *0.209* | *51.744* | *0.217* |
| ***N*** | *17646* | | *15804* | |

*^a^ Father’s and adult social class is a ridit score from 0 to 1 with a value closer to 1 indicating more disadvantaged social class. Analysis carried out using linear regression.*

*** p<0.001 *p<0.05*
