# Supplementary material for: Childhood socioeconomic position and adult mental wellbeing: Evidence from four British birth cohort studies
Source: PLoS One. 2017 Oct 25;12(10):e0185798. doi: 10.1371/journal.pone.0185798 (PMC5656308; doi:10.1371/journal.pone.0185798)
Supplement: S2 Table — (DOCX) [file pone.0185798.s002.docx]

S2 Table: Sex-adjusted associations between Warwick-Edinburgh Mental Well-Being scores and father's social class or adult social class coded as categorical variables

|  | **HCS** | | **NSHD** | | **NCDS** | | **BCS70** | |
| --- | --- | --- | --- | --- | --- | --- | --- | --- |
|  | **Coef.** | **SE** | **Coef.** | **SE** | **Coef.** | **SE** | **Coef.** | **SE** |
| **Father's social class (ref: III Skilled Manual)** |  |  |  |  |  |  |  |  |
| I (Professional) | 1.160 | 2.075 | 0.956 | 0.804 | 1.116 | 0.440 | 1.747 | 0.439 |
| II (Intermediate) | 0.284 | 0.816 | 0.677 | 0.560 | 0.801 | 0.252 | 1.748 | 0.245 |
| III (Skilled non-manual) | -0.522 | 0.801 | 0.408 | 0.599 | 0.929 | 0.316 | 1.173 | 0.329 |
| IV (Semi-skilled manual) | -0.759 | 0.536 | -0.164 | 0.596 | -0.085 | 0.291 | 0.238 | 0.310 |
| V (Unskilled) | -2.174 | 0.804 | -0.085 | 0.973 | -0.297 | 0.372 | -0.598 | 0.431 |
|  |  | |  | |  | |  | |
| **Adult social class (ref: II Intermediate)** |  |  |  |  |  |  |  |  |
| I (Professional) | 0.604 | 0.902 | 0.078 | 0.772 | -0.032 | 0.395 | 0.325 | 0.381 |
| III (Skilled Non-Manual) | -0.499 | 0.748 | -1.535 | 0.513 | -1.532 | 0.240 | -1.680 | 0.252 |
| III (Skilled Manual) | -0.289 | 0.551 | -2.456 | 0.576 | -1.649 | 0.251 | -1.836 | 0.268 |
| IV (Partly Skilled) | -0.471 | 0.717 | -2.593 | 0.689 | -2.432 | 0.289 | -1.760 | 0.297 |
| V (Unskilled) | 0.707 | 1.346 | -2.578 | 1.215 | -3.563 | 0.521 | -3.649 | 0.660 |

*Note that departure from non-linearity in the association between wellbeing and social class was tested using the likelihood ratio test. There was no evidence of difference in goodness of fit on comparison of models with social class entered as categorical versus continuous variables*
