# Supplementary material for: Childhood socioeconomic position and adult mental wellbeing: Evidence from four British birth cohort studies
Source: PLoS One. 2017 Oct 25;12(10):e0185798. doi: 10.1371/journal.pone.0185798 (PMC5656308; doi:10.1371/journal.pone.0185798)
Supplement: S3 Table — (DOCX) [file pone.0185798.s003.docx]

S3 Table: Gender-adjusted associations between adult mental wellbeing and social class in childhood and adulthood

|  | **Father’s social class (model 1)** | | **Adult social class  (model 2)** | | **Father's social class and adult social class  (model 3)** | |
| --- | --- | --- | --- | --- | --- | --- |
|  | **Coeff** | **SE** | **Coeff** | **SE** | **Coeff** | **SE** |
| **Father's social class (ridit score)^a^** | -2.768^**^ | 0.346 |  |  | -2.121^**^ | 0.353 |
| **Cohort*father's social class (ref: BCS70*father's social class) ^a^** |  |  |  |  |  |  |
| NCDS | 1.147^*^ | 0.496 |  |  | 1.310^*^ | 0.507 |
| NSHD | 1.482 | 0.800 |  |  | 2.217^*^ | 0.845 |
| HCS | 0.823 | 0.905 |  |  | 0.192 | 0.919 |
| **Adult social class (ridit score)^a^** |  |  | -3.588^**^ | 0.355 | -3.274^**^ | 0.363 |
| **Cohort*adult social class (ref: BCS70*adult social class) ^a^** |  |  |  |  |  |  |
| NCDS |  |  | -0.113 | 0.489 | -0.298 | 0.502 |
| NSHD |  |  | -0.525 | 0.817 | -0.815 | 0.866 |
| HCS |  |  | 2.884^*^ | 0.891 | 2.965^*^ | 0.908 |
| **Cohort (ref: BCS70)** |  |  |  |  |  |  |
| NCDS | -0.503 | 0.281 | 0.142 | 0.278 | -0.449 | 0.339 |
| NSHD | 1.724^**^ | 0.453 | 2.712^**^ | 0.456 | 1.719^*^ | 0.530 |
| HCS | 2.356^**^ | 0.512 | 1.327^*^ | 0.509 | 1.159 | 0.625 |
| **Sex (ref: male)** |  |  |  |  |  |  |
| Female | 0.063 | 0.114 | 0.140 | 0.114 | 0.140 | 0.113 |
| **Partnership (ref: partnered)** |  |  |  |  |  |  |
| Unpartnered |  |  |  |  |  |  |
| **Long-term limiting illness (ref: no)** |  |  |  |  |  |  |
| Yes |  |  |  |  |  |  |
| *Constant* | *50.500* | *0.207* | *50.874* | *0.210* | *51.809* | *0.251* |

*^a^ Father’s and adult social class is a ridit score from 0 to 1 with a value closer to 1 indicating more disadvantaged social class. Analysis carried out using linear regression.*

*** p<0.001 *p<0.05*
