# Supplementary material for: Childhood socioeconomic position and adult mental wellbeing: Evidence from four British birth cohort studies
Source: PLoS One. 2017 Oct 25;12(10):e0185798. doi: 10.1371/journal.pone.0185798 (PMC5656308; doi:10.1371/journal.pone.0185798)
Supplement: S4 Table — (DOCX) [file pone.0185798.s004.docx]

S4 Table: Comparison of the cases with missing data on the Warwick-Edinburgh Mental Well-Being Scale and cases with complete data on the Warwick-Edinburgh Mental Well-Being Scale

|  | **HCS** | | **NSHD** | | **NCDS** | | **BCS70** | |
| --- | --- | --- | --- | --- | --- | --- | --- | --- |
|  | **Missing data on WEMWBS** | **Complete data on WEMWBS** | **Missing data on WEMWBS** | **Complete data on WEMWBS** | **Missing data on WEMWBS** | **Complete data on WEMWBS** | **Missing data on WEMWBS** | **Complete data on WEMWBS** |
|  | **%** | **%** | **%** | **%** | **%** | **%** | **%** | **%** |
| **Male** | 64.3 | 50.7 | 53.2 | 47.0 | 58.3 | 48.2^**^ | 54.7 | 47.0^**^ |
| **Father's social class** |  |  |  |  |  |  |  |  |
| I (Professional) | 0.0 | 1.1 | 3.1 | 7.7^*^ | 3.1 | 5.2^*^ | 4.2 | 5.5^*^ |
| II (Intermediate) | 16.7 | 8.5 | 22.3 | 21.3 | 18.4 | 21.3^*^ | 21.3 | 26.1^**^ |
| III (Skilled Non-Manual) | 8.3 | 8.9 | 15.6 | 17.2 | 9.0 | 11.3^*^ | 10.9 | 11.1 |
| III (Skilled Manual) | 41.7 | 46.9 | 32.1 | 31.6 | 44.5 | 40.5 | 43.3 | 38.4 |
| IV (Partly Skilled) | 33.3 | 25.8 | 21.4 | 17.4 | 14.4 | 14.1 | 12.8 | 13.0 |
| V (Unskilled) | 0.0 | 8.8 | 5.4 | 4.9 | 10.6 | 7.6 | 7.6 | 5.8 |
| **Mean years of education** | 15.4 | 15.5 | 16.8 | 17.0 | 17.9 | 18.4^*^ | 17.9 | 18.3^*^ |
| **Adult social class** |  |  |  |  |  |  |  |  |
| I (Professional) | 7.1 | 7.4 | 4.5 | 7.5 | 5.1 | 5.6 | 4.0 | 6.4^*^ |
| II (Intermediate) | 21.4 | 27.7 | 41.5 | 41.1 | 32.9 | 38.5 | 41.6 | 44.9 |
| III (Skilled Non-Manual) | 7.1 | 12.2 | 22.8 | 23.7 | 19.3 | 21.9 | 18.3 | 18.8 |
| III (Skilled Manual) | 42.9 | 36.0 | 17.9 | 15.5 | 24.5 | 18.7^**^ | 21.7 | 16.2^**^ |
| IV (Partly Skilled) | 14.3 | 13.8 | 11.2 | 9.6 | 14.3 | 12.2^*^ | 12.9 | 11.8 |
| V (Unskilled) | 7.1 | 2.9 | 2.2 | 2.6 | 3.9 | 3.0^*^ | 1.6 | 2.0 |
| ***Total*** | *14* | *1,402* | *248* | *1,978* | *1,045* | *8,745* | *1,252* | *8,589* |

** p<0.05 **p<0.001 missing data v complete data.*

*Statistical comparisons carried out using logistic regression modelling likelihood of missingness as a function of gender (reference men), social class (reference skilled manual) or education (per additional year)*
