# Supplementary material for: Childhood socioeconomic position and adult mental wellbeing: Evidence from four British birth cohort studies
Source: PLoS One. 2017 Oct 25;12(10):e0185798. doi: 10.1371/journal.pone.0185798 (PMC5656308; doi:10.1371/journal.pone.0185798)
Supplement: S5 Table — (DOCX) [file pone.0185798.s005.docx]

S5 Table: Childhood social class and adult social class associations with the Warwick-Edinburgh Mental Well-Being scores, complete cases

|  | **Childhood social class**  **(model 1)** | | **Adult social class**  **(model 2)** | | **Childhood social class and adult social class**  **(model 3)** | |
| --- | --- | --- | --- | --- | --- | --- |
|  | **Coef** | **SE** | **Coef** | **SE** | **Coef** | **SE** |
| **Father's social class^a^ (ridit score)** | -1.665^**^ | 0.355 |  |  | -1.174^*^ | 0.357 |
| **Cohort*father's social class (ref: BCS70*father's social class)** |  |  |  |  |  |  |
| NCDS | 0.553 | 0.502 |  |  | 0.649 | 0.500 |
| NSHD | 0.693 | 0.838 |  |  | 0.989 | 0.836 |
| HCS | 0.118 | 0.878 |  |  | 0.131 | 0.874 |
| **Adult social class^a^ (ridit score)** |  |  | -2.916^**^ | 0.361 | -2.541^**^ | 0.232 |
| **Cohort*adult social class (ref: BCS70*adult social class)** |  |  |  |  |  |  |
| NCDS |  |  | 0.003 | 0.497 |  |  |
| NSHD |  |  | -0.497 | 0.853 |  |  |
| HCS |  |  | 2.952^*^ | 0.856 |  |  |
| **Cohort (ref: BCS70)** |  |  |  |  |  |  |
| NCDS | -0.989^*^ | 0.287 | -0.704^*^ | 0.287 | -1.033^**^ | 0.286 |
| NSHD | 1.601 | 0.478 | 2.174^**^ | 0.483 | 1.432^*^ | 0.477 |
| HCS | 2.373 | 0.499 | 0.945 | 0.489 | 2.348^**^ | 0.498 |
| **Sex (ref: male)** |  |  |  |  |  |  |
| Female | 0.263^*^ | 0.125 | 0.317^*^ | 0.125 | 0.309^*^ | 0.125 |
| **Partnership (ref: partnered)** |  |  |  |  |  |  |
| Unpartnered | -1.845^**^ | 0.163 | -1.752^**^ | 0.162 | -1.744^**^ | 0.162 |
| **Long-term limiting illness (ref: no)** |  |  |  |  |  |  |
| Yes | -3.033^**^ | 0.160 | -2.988^**^ | 0.160 | -2.981^**^ | 0.160 |
| ***Constant*** | *51.584* | *0.218* | *52.162* | *0.221* | *52.567* | *0.235* |
| ***N*** | *15,366* | | | | | |

*^a^ Father’s and adult social class is a ridit score from 0 to 1 with a value closer to 1 indicating more disadvantaged social class. Statistical analysis carried out using linear regression.*

*** p<0.001 *p<0.05.*
